# Supplementary material for: Arduino Automated Microwave Oven for Tissue Decalcification
Source: Bioengineering (Basel). 2023 Jan 6;10(1):79. doi: 10.3390/bioengineering10010079 (PMC9855053; doi:10.3390/bioengineering10010079)
Supplement: Supplementary file 1 [file bioengineering-10-00079-s001.zip › bioengineering-2007630-supplementary.pdf]

bioengineering

MDPI

Article

Arduino Automated Microwave Oven for Tissue Decalcification

Paolo Savadori [1,2,*], Sophia Dalfino [1,3], Marco Piazzoni [1,4], Francesco Inchingolo [5], Massimo Del Fabbro [1,2], Gianluca Martino Tartaglia [1,2] and Luciano Giardino [6]

1 Department of Biomedical, Surgical and Dental Sciences, Università degli Studi di Milano, 20133 Milan, Italy
2 IRCCS Fondazione Ca'Granda IRCCS Ospedale Maggiore Policlinico, 20122 Milan, Italy
3 Complex Tissue Regeneration Department, MERLN Institute for Technology-Inspired Regenerative Medicine, 6229 Maastricht, The Netherlands
4 C. Advanced Biomaterials Lab, Physics Department, Università degli Studi di Milano, 20133 Milano, Italy
5 Interdisciplinary department of Medicine, Università di Bari "Aldo Moro", 70121 Bari, Italy
6 Freelance Researcher; 88900 Crotone, Italy
* Correspondence: paolo.savadori@unimi.it (P.S.)

Supplementary Materials

Table S1. Arduino sketch.

```
// constants won't change
const int RELAY_PIN = 3;  // the Arduino pin, which
connects to the IN pin of relay

// the setup function runs once when you press reset or
power the board
void setup() {
  // initialize digital pin as an output.
  pinMode(RELAY_PIN, OUTPUT);
}

// the loop function runs over and over again forever
void loop() {
  int i = 0;
  while ( i < 20000) {
    digitalWrite(RELAY_PIN, HIGH);
    delay(1);
    digitalWrite(RELAY_PIN, LOW);
    delay(1);
    i++;
  }
  digitalWrite (RELAY_PIN, LOW);
  delay(3600000);
}
```

Table S2. Modified hematoxylin and eosin protocol.

Hydration	Xylene	15 minutes
	Ethanol 100%	5 minutes
	Ethanol 90%	5 minutes
	Ethanol 70%	5 minutes
	Distilled water	5 minutes
Nuclear staining	Morel and Bassal hematoxylin	15 minutes
	Composition:	
	Solution A	
	Hematoxylin	1gr
	Ethanol 95%	100ml
	Solution B	
	Ferric chloride	2gr
	Cupric acetate	0.04gr
	Hydrochloric acid 37%	1ml
	Mix solution A and solution B 1:1	
	Wash with distilled water	
Differentiation	Acidified Ethanol	5 seconds
	Composition: Ethanol	70%
	HCl	1%
	Wash with distilled water	
Bluening	Ammonium Hydroxide 1%	30 seconds
	Wash with distilled water	
	Ethanol 80%	5 minutes
Counterstaining	Trichrome eosin	5 minutes
	Composition:	
	Eosin 1% (H_20)	20ml
	Phloxin B 1% (H_20)	2ml
	Orange G 1% (H_20)	2ml
	Glacial acetic acid	0.8ml
	Ethanol 95%	156ml
	Wash with distilled water	
dehydration	Ethanol 100%	5 minutes
	Ethanol 100%	5 minutes
	Xylene 100%	5 minutes
	Xylene 100%	5 minutes
	Mount the slide	
